# Reduced Risk of Atrial Fibrillation Following Cholecystectomy: A Nationwide Population-Based Study

**DOI:** 10.3389/fnagi.2021.706815

**Published:** 2021-09-02

**Authors:** Tung Ching Ho, Yu-Ching Chen, Che-Chen Lin, Hsu-Chih Tai, Cheng-Yu Wei, Yung-Hsiang Yeh, Chung Y. Hsu

**Affiliations:** ^1^Department of Cardiology, Chang Hua Hospital, Changhua County, Taiwan; ^2^Department of Bioinformatics and Medical Engineering, College of Information and Electrical Engineering, Asia University, Taichung, Taiwan; ^3^Management Office for Health Data, China Medical University Hospital, Taichung, Taiwan; ^4^Department of Exercise and Health Promotion, College of Kinesiology and Health, Chinese Culture University, Taipei, Taiwan; ^5^Department of Neurology, Chang Bing Show Chwan Memorial Hospital, Changhua County, Taiwan; ^6^Digestive Disease Center, Chang Bing Show Chwan Memorial Hospital, Changhua County, Taiwan; ^7^Graduate Institute of Clinical Medicine Science and School of Medicine, College of Medicine, China Medical University, Taichung, Taiwan

**Keywords:** atrial fibrillation, risk factors, gallstone disease, cholelithiasis, cholecystectomy, prevention

## Abstract

**Background**: Gallstone disease (GD) is associated with a high risk of cardiovascular disease. However, it is unknown whether GD contributes to atrial fibrillation (AF). We aimed to investigate the association between GD and AF.

**Methods**: We performed a population-based cohort study using data from the Taiwan National Health Insurance Research Database between 2001 and 2011. A GD cohort of 230,076 patients was compared with a control cohort consisting of an equal number of patients matched for age, sex, cardiovascular and gastrointestinal comorbidities.

**Results**: In total, 5,992 (49.8/10,000 person-years) patients with GD and 5,804 (44.5/10,000 person-years) controls developed AF. GD increased AF risk with a hazard ratio (HR) of 1.20 [95% confidence interval (CI), 1.16–1.25]. In patients with GD but without cholecystectomy, the HR of AF reached 1.57 (95% CI = 1.50–1.63). After cholecystectomy, the HR of AF significantly decreased to 0.85 (95% CI = 0.81–0.90). Among the three age groups with GD (<45, 45–64, and ≥65 years), the adjusted HRs of AF were 1.59 (95% CI = 1.08–2.33), 1.31 (95% CI = 1.18–1.45), and 1.18 (95% CI = 1.13–1.22), respectively. Compared with patients with a CHA_2_DS_2_-VASc score equal to 0, the HRs of AF risk among total cohort patients and a score equal to 1, 2, 3, and ≥ 4 were 1.28 (95% CI = 1.15–1.43), 2.26 (95% CI = 2.00–2.56), 3.81 (95% CI = 3.35–4.34), and 5.09 (95% CI = 4.42–5.87), respectively.

**Conclusion**: This population-based longitudinal follow-up study showed that patients with GD had an increased AF risk. Moreover, cholecystectomy was related to reduced AF risk. Cardiovascular checkups may be necessary for patients with GD, especially those who are young and have other typical risk factors.

## Introduction

Atrial fibrillation (AF) is the most common sustained cardiac arrhythmia in adults. Although AF prevalence varies among ethnic populations, its prevalence progressively increases with advancing age. It occurs in <1% of individuals aged 60–65 years but in 8% to 10% of individuals aged >80 years (Kannel and Benjamin, 2009; Rodriguez et al., 2015). AF can lead to acute or chronic severe outcomes, including embolic stroke, extracranial systemic thromboembolism, dementia, heart failure (HF), myocardial infarction, and venous thromboembolism (Staerk et al., 2017). Patients with AF may experience various symptoms such as palpitations, shortness of breath, exercise intolerance, chest pain, fatigue, dizziness, and light-headedness (Reynolds et al., 2006; Zimetbaum, 2017). However, up to 90% of AF events may be symptomless, particularly in the elderly (Page et al., 1994). Therefore, early recognition and diagnosis of AF are crucial, particularly in people with risk factors (RFs).

Some unmodifiable and modifiable RFs are well established for AF (Staerk et al., 2017). The unmodifiable RFs include genetics age gender, and race (Marcus et al., 2010; Rodriguez et al., 2015). The modifiable RFs include physical inactivity, smoking, obesity, diabetes mellitus (DM), obstructive sleep apnea (OSA), and hypertension (Alonso et al., 2013; Schnabel et al., 2015). However, the mechanisms that cause AF are unclear. AF may occur in the absence of known structural or electrophysiological abnormalities (Staerk et al., 2017). Epidemiological studies have revealed many potential RFs for AF. In the field of digestive disorders, gastroesophageal reflux disease (GERD; Huang et al., 2012), inflammatory bowel disease (IBD; Choi et al., 2019), gastrointestinal cancers (Jakobsen et al., 2019), and liver disease (Lee et al., 2017; Huang et al., 2018) have been reported to be associated with significantly increased AF risk.

Gallstone disease (GD; cholelithiasis) is a common digestive disorder and significantly increases cardiovascular disease (CVD) risk (Bortnichak et al., 1985; Jiang et al., 2013; Olaiya et al., 2013; Wei et al., 2014, 2019; Lv et al., 2015; Wirth et al., 2015; Zheng et al., 2016, 2018; Fan et al., 2017; Upala et al., 2017; Fairfield et al., 2019). However, no studies on AF risk in patients with GD have been conducted. Therefore, we investigated the relationship between GD and later AF development by using a prospective, nationwide, and case-cohort study design. If gallbladder removal reduces AF incidence, it would serve as evidence for GD being a risk factor for AF. Therefore, we further compared patients with GD who did not undergo cholecystectomy with those who underwent cholecystectomy.

## Materials and Methods

### Data Source

The Taiwan National Health Insurance program is a universal, single-payer health insurance program covering 99% of the residents of Taiwan. The Taiwan government has transferred medical claims data to the National Health Research Institutes (NHRI), which has established and managed a database named the National Health Insurance Research Database (NHIRD). The NHIRD contains medical claims data, including a registry of beneficiaries, disease record files, and other medical services. The NHIRD uses the *International Classification of Diseases, Ninth Revision, Clinical Modification* (*ICD-9-CM*) for coding diseases. The NHRI has released the database (for research use) and assigned a unique encoded number for accessing the annual medical history of each insured individual. Moreover, this study was approved by the Ethics Review Board of China Medical University (CMUH104-REC2-115).

### Study Population

**Figure 1:**
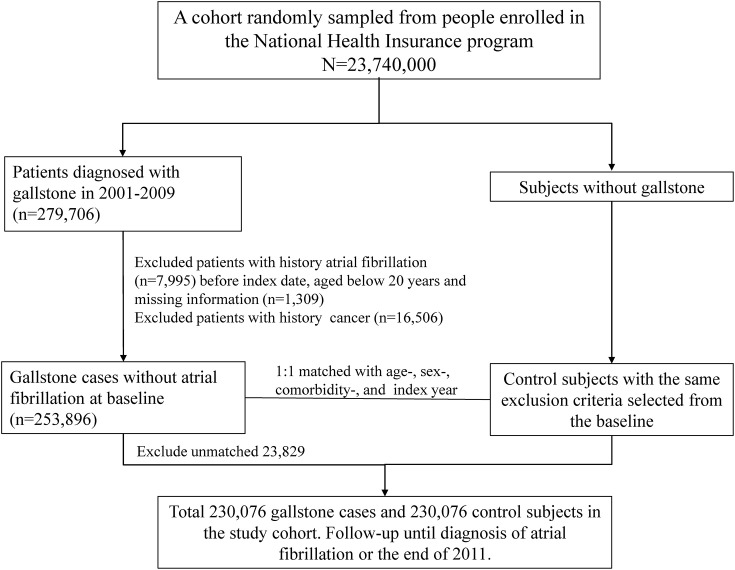
Flowchart for study population selection.

This study investigated the AF risk in patients with GD. This study had two stages. We established a GD cohort and a control cohort to observe AF occurrence. The GD cohort involved aged ≥20 years patients from January 1, 2001, to December 31, 2009, and assigned the index date as the first date of newly diagnosed GD (*ICD-9-CM* 574), which included gallbladder stones (cholecystolithiasis), intrahepatic bile duct stones (hepatolithiasis) and extrahepatic bile duct stones (choledocholithiasis; Lammert et al., 2016). The GD cohort was further subgrouped in asymptomatic (*ICD-9-CM* 574.2, 574.5, and 574.9) and symptomatic GD (*ICD-9-CM* 574.0, 574.1, 574.3, 574.4, 574.6, 574.7, and 574.8). The patients with symptomatic GD are meant to have specific or nonspecific symptoms or complications, such as biliary colic, intolerance to fatty foods, and infections (Lammert et al., 2016). Furthermore, we categorized GD into two subgroups: with and without cholecystectomy. An equal number of control individuals without GD were randomly selected by using the propensity matching method. The matching criteria were age, sex, cardiovascular and gastrointestinal comorbidities. The cardiovascular comorbidities included hypertension (*ICD-9-CM* 401–405), DM (*ICD-9-CM* 250), coronary artery disease (CAD, *ICD-9-CM* 410–414), chronic kidney disease (CKD, *ICD-9-CM* 585, 586, 588.8, and 588.9), hyperlipidemia (*ICD-9-CM* 272), and HF (*ICD-9-CM* 428). The gastrointestinal comorbidities included GURD (*ICD-9-CM* 533, 530.11, and 530.81), IBD (*ICD-9-CM* 555, 556), hepatitis C (*ICD-9-CM* 070.41, 070.44, 070.51, 070.54, 070.70, 070.71, and V02.62), and nonalcoholic fatty liver disease (NAFLD, *ICD-9-CM* 571.8). We assigned the index date for control individuals as a random month and day but with the same index year as the matched cases. The GD and control cohorts excluded individuals without AF diagnosis (*ICD-9-CM* 427.31) and various kinds of cancer (*ICD-9-CM* 140–239) before the index date. Follow-up ceased when an individual withdrew from the insurance program, an AF event occurred, or the date reached December 31, 2011 (Figure 1).

CHA_2_DS_2_-VASc [Congestive heart failure, Hypertension, Age ≥75 years (doubled), Diabetes, prior Stroke or transient ischemia attack (doubled)—Vascular disease, Age 65–74 years, and Sex category (female)] was used for predicting AF in patients with GD. The score was calculated for each patient by assigning 1 point for HF, hypertension, DM, vascular disease (*ICD-9-CM* 410, 412, 420.20, 420.21, 420.22, 420.23, 420.24, 420.29, 429.79, 440.0, 440.20, 440.21, 440.22, 440.23, 440.24, 440.3, 440.4, 443.81, 443.89, and 443.9), sex category (female), and age (65–74 years) and 2 points for any history of ischemic stroke or transient ischemia attack (*ICD-9-CM* 433–438) and age ≥75 years (Lip et al., 2010; Camm et al., 2012; January et al., 2014).

### Statistical Analysis

To present the distribution of the study cohorts, we indicate the number and percentage for all the category variables (including age group, sex, and comorbidities). To assess the distribution difference between the two cohorts, we used the standard mean difference. The incidence destiny of AF for each cohort was calculated as the number of patients with AF divided by the total number of patients followed up. The cumulative incidence curve was measured using the Kaplan–Meier method, and the curve difference was tested using the log-rank test. To analyze the difference in AF risk between the GD cohort and the control cohort, we estimated the hazard ratios (HRs) and 95% confidence intervals (CIs) by using the crude and adjusted Cox proportional hazard models. Moreover, we used Cox models to determine the influence of cholecystectomy and various GD subtypes on AF risk. Furthermore, we applied a subgroup analysis for comparing patients with GD with control individuals through the stratification of variables, including age group, sex, and comorbidities. Additionally, Cox models were used to estimate AF risk associated with CHA_2_DS_2_-VASc score. SAS 9.4 software (SAS Institute, Cary, NC, USA) was used to perform the analyses, and R software (R Foundation for Statistical Computing, Vienna, Austria) was used to determine the incidence curves. The significance level was set at *p* < 0.05 for 2-sided testing.

## Results

This study involved two cohorts: a cohort of 230,076 patients with GD and a control cohort consisting of an equal number of healthy individuals (Table 1). In the study cohort, the majority of the participants were ≥65 years old (approximately 43%), and the proportions of men and women were approximately equal. Furthermore, the comorbidities, including hypertension, DM, CAD, CKD, hyperlipidemia, HF, GURD, IBD, hepatitis C, and NAFLD, were similar between the GD cohort and the control cohort.

**Table 1:** Demographic characteristics and comorbidities in GD and control groups.

Variable	Control cohort N = 230076 (%)	Gallstone cohort N = 230076 (%)	Standard mean difference^§^
Age group, years			
< 45	50,821 (22.1)	50,634 (22.0)	0.002
45–64	80,433 (35.0)	82,529 (35.9)	0.02
≥65	98,822 (43.0)	96,913 (42.1)	0.017
Mean (SD)	59.9 (17.0)	59.6 (16.8)	0.01
Sex			
Female	117,990 (51.3)	116,694 (50.7)	0.01
Male	112,086 (48.7)	11,382 (49.3)	0.01
Cardiovascular comorbidity			
Hypertension	46,670 (20.3)	44,124 (19.2)	0.03
DM	29,727 (12.9)	27,762 (12.1)	0.03
CAD	22,315 (9.70)	20,887 (9.08)	0.02
CKD	3,726 (1.62)	3,756 (1.63)	0.001
Hyperlipidemia	11,840 (5.15)	11,222 (4.88)	0.01
HF	6,263 (2.72)	7,036 (3.06)	0.02
Gastrointestinal comorbidity			
GURD	13,598 (5.91)	14,536 (6.32)	0.02
IBD	295 (0.17)	411 (0.18)	0.002
Hepatitis C	2,216 (0.96)	2,916 (1.27)	0.03
NAFLD	8,290 (3.60)	8,082 (3.51)	0.005

*GD, gallstone disease; DM, diabetes mellitus; CAD, coronary artery disease; CKD, chronic kidney disease; HF, heart failure; GURD, gastroesophageal reflux disease; IBS, inflammatory bowel; NAFLD, nonalcoholic fatty liver disease ^§^Standard mean difference ≤0.10 indicates a negligible difference between the two cohorts*.

**Figure 2:**
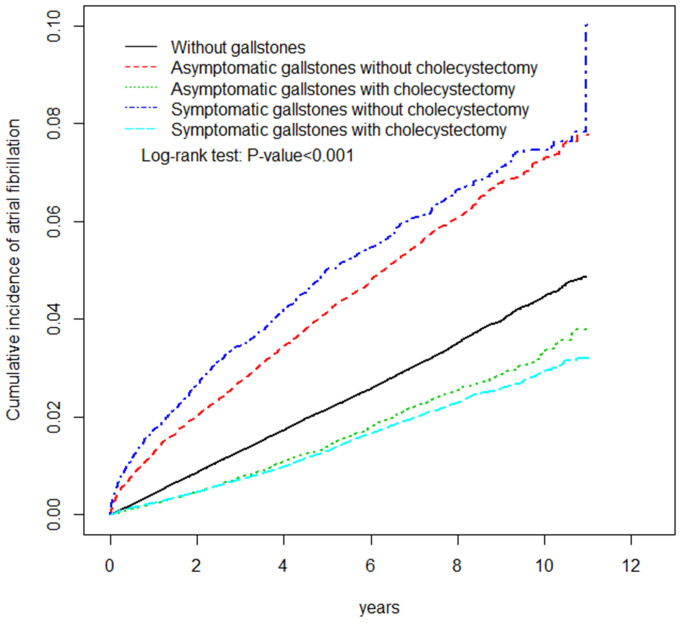
The adjusted cumulative incidence curves of atrial fibrillation between patients with or without gallstone disease.

AF occurred in 5,804 and 5,992 participants in the control and GD cohorts, respectively (Table 2). The AF incidences were 44.5 and 49.8 per 10,000 person-years in the control and GD cohorts, respectively. Figure 2 shows that the cumulative incidence curves of AF were significantly higher in the GD without cholecystectomy cohort and lower in the GD with cholecystectomy than in the control cohort (log-rank test, *p* < 0.001). After adjusting for age, sex, and comorbidities, the GD group was significantly associated with increased AF risk compared with the control group (HR = 1.20, 95% CI = 1.16–1.25). Moreover, Table 2 shows that relative to the control cohort, the patients with GD who did not undergo cholecystectomy had a 1.57-fold increased AF risk (95% CI = 1.50–1.63), whereas patients with GD who underwent cholecystectomy had a 0.85-fold decreased AF risk (95% CI = 0.81–0.90). Simultaneously, compared with the patients with GD and without cholecystectomy, the HR of AF for patients with GD who underwent cholecystectomy was 0.54 (95% CI = 0.51–0.57). The incidence and HR of AF were calculated according to various GD subtypes. Compared with the patients without GD, those with asymptomatic and symptomatic GD had 1.33- and 1.07-fold increased AF risk (95% CI = 1.27–1.39 and 1.02–1.12), respectively.

**Table 2:** Incidence and HRs of AF in GD and control groups.

GD treatment	*N*	Event	PYs	Rate	HRs	
					Model 1	Model 2
Without GD	230,076	5,804	130,5065	44.5	ref	
With GD	230,076	5,992	120,3452	49.8	1.20 (1.16, 1.25)***	
GD without cholecystectomy	97,408	3,814	440,837	86.5	1.57 (1.50, 1.63)***	ref
GD with cholecystectomy	132,668	2,178	762,615	28.6	0.85 (0.81, 0.90)***	0.54 (0.51, 0.57)***
Asymptomatic GD	107,561	3,396	518,529	65.5	1.33 (1.27, 1.39)***	
Symptomatic GD	122,515	2,596	684,898	37.9	1.07 (1.02, 1.12)**	

Model 1 and model 2 adjusted for age, sex, cardiovascular, and gastrointestinal comorbidities. AF, atrial fibrillation; PYs, person-years; Rate, incidence rate, per 10,000 person-years; HRs, hazard ratios; ref, reference. **p < 0.01, ***p < 0.0001.

**Figure 3:**
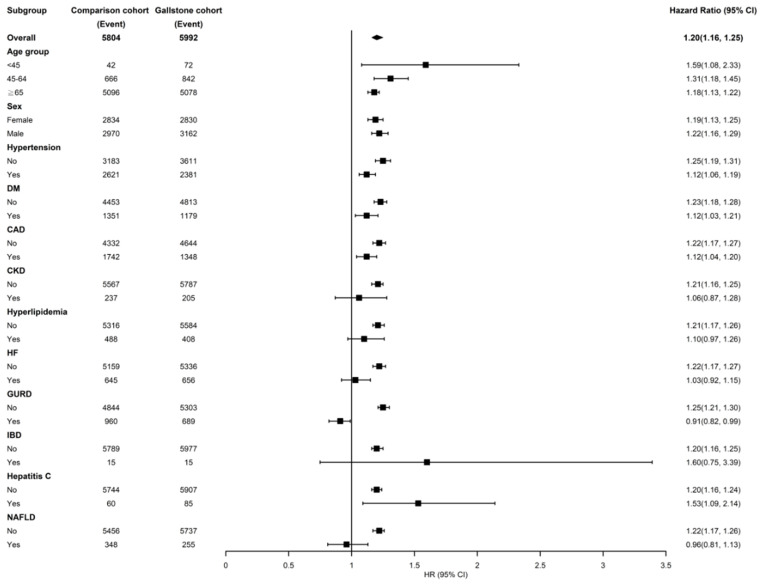
Incidence and hazard ratios of atrial fibrillation stratified by age, sex, and comorbidities in the patients of gallstone disease. DM, diabetes mellitus; CAD, coronary artery disease; CKD, chronic kidney disease; HF, heart failure; GURD, gastroesophageal reflux disease; IBS, inflammatory bowel; NAFLD, nonalcoholic fatty liver disease.

Figure 3 shows the stratified analysis for patients with GD and the control cohort. Relative to the control cohort, the adjusted HRs of AF were 1.59 (95% CI = 1.08–2.33), 1.31 (95% CI = 1.18–1.45), and 1.18 (95% CI = 1.13–1.22) for patients with GD in the age groups of < 45, 45–64, and ≥65 years, respectively. The female and male patients with GD had a 1.19- and 1.22-fold increased AF risk compared with female (95% CI = 1.13–1.25) and male (95% CI = 1.16–1.29) control individuals, respectively. Among the noncomorbid patients, those with GD had a significantly higher AF risk than those in the control cohort (HRs for hypertension, DM, CAD, CKD, hyperlipidemia, HF, GURD, IBD, hepatitis C, and NAFLD were 1.25, 1.23, 1.22, 1.21, 1.21, 1.22, 1.25, 1.20, 1.20 and 1.22 respectively).

Table 3 shows the incidence and HRs of AF in three study cohorts stratified based on CHA_2_DS_2_-VASc score. In total study cohort patients, including GD and non-GD patients, the incidence rate of AF increased from 9.08 per 10,000 person-years for patients with a CHA_2_DS_2_-VASc score of 0 up to 172.1 per 10,000 person-years for those with a CHA_2_DS_2_-VASc score of at least 4. The HR of AF was 5.09 (95% CI = 4.42–5.84) for those with a CHA_2_DS_2_-VASc score of at least 4 as compared with those with a CHA_2_DS_2_-VASc score of 0. In GD patients with and without cholecystectomy, the incidence rates and HRs were also gradually increased as the CHA2DS2-VASc score increased from 0 to at least 4.

**Table 3:** Incidence and HRs of AF in study cohort patients stratified by CHA_2_DS_2_-VASc score.

CHA_2_DS_2_-VASc	*N*	Event	PYs	Rate	HR (95% CI)
Total study cohort patients					
0	106,900	579	637,602	9.08	ref
1	163,602	1,533	986,007	15.6	1.28 (1.15, 1.43)***
2	73,853	2,670	393,492	67.9	2.26 (2.00, 2.56)***
3	51,643	2,774	245,102	113.2	3.81(3.35, 4.34)***
≥4	64,154	4,240	246,313	172.1	5.09 (4.42, 5.87)***
GD without cholecystectomy					
0	87,484	386	533,446	7.24	ref
1	139,938	1,106	857,101	12.9	1.35 (1.19, 1.54)***
2	56,059	1,908	314,054	60.8	2.46 (2.12, 2.85)***
3	36,708	1,867	186,091	100.3	4.09 (3.49, 4.81)***
≥4	42,555	2,715	176,987	153.4	5.53 (4.64, 6.59)***
GD with cholecystectomy					
0	19,416	193	104,156	18.5	ref
1	23,664	427	128,906	33.1	1.14 (0.94, 1.39)
2	17,794	762	79,438	95.9	1.69 (1.36, 2.08)***
3	14,935	907	59,011	153.7	2.63 (2.11, 3.29)***
≥4	21,599	1,525	69,326	220.0	3.17 (2.49, 4.03)***

HRs, hazard ratios; AF, atrial fibrillation; GD, gallstone disease; PYs, person-years; Rate, incidence rate, per 10,000 person-years; HRs, hazard ratios; ref, reference. ***p < 0.0001.

## Discussion

This study demonstrated that GD was independently associated with an increased AF risk in a population-based cohort, suggesting that GD may play a crucial role in determining AF risk. Furthermore, we found that patients with GD who underwent cholecystectomy were 0.85- and 0.54-fold less likely to develop AF compared with individuals without GD and patients with GD who did not undergo cholecystectomy, respectively. This result may provide further evidence that GD is one of the potential RFs for AF. GD is a common disorder that affects approximately 10%-20% of the global adult population (Lammert et al., 2016). Simultaneously, GD and AF share several RFs, including age, physical inactivity, obesity, and DM (Lammert et al., 2016; Staerk et al., 2017; Ibrahim et al., 2018). Many original and meta-analysis studies have highlighted the importance of recognizing GD as a risk factor for CVD and stroke (Bortnichak et al., 1985; Jiang et al., 2013; Olaiya et al., 2013; Wei et al., 2014, 2019; Lv et al., 2015; Wirth et al., 2015; Zheng et al., 2016, 2018; Fan et al., 2017; Upala et al., 2017; Fairfield et al., 2019). AF is known to be a major cause of systemic embolism and stroke (Staerk et al., 2017), but the association between GD and AF has remained unclear. This is the first longitudinal follow-up study to reveal that GD increases AF risk.

In the study, both asymptomatic and symptomatic GD without gallbladder removal exhibited a high AF risk. In fact, 80% of the patients with GD were asymptomatic, but these patients exhibited approximately a 1%-3% annual and 7%-26% lifetime risk of developing complications related to biliary colic or gallstones (Friedman et al., 1989; Friedman, 1993; Ibrahim et al., 2018). Conventionally, expectant management is the standard treatment for asymptomatic GD, except for patients with chronic hemolytic anemia or who are Native Americans (Ibrahim et al., 2018). Our previous findings have suggested that patients with GD have a high risk of stroke, but cholecystectomy reduces the risk of ischemic and hemorrhagic stroke among both asymptomatic and symptomatic patients (Wei et al., 2014, 2019). Therefore, patients with GD should receive frequent cardiovascular checkups. In addition, GD and AF must be studied in further detail.

In the stratified analysis based on age (45, 45–64, and ≥65 years), GD exhibited an obvious association with AF. This suggests that attention should be paid to preventing embolic stroke, particularly in young GD patients. Although epidemiological data have shown a high prevalence of GD and low prevalence of AF in women (Lammert et al., 2016; Staerk et al., 2017), both female and male patients with GD had an obvious risk of AF in the study. The GD groups had a significantly higher AF risk than the control groups without comorbidities. Thus, GD might be a newfound risk factor for AF.

Although CHA_2_DS_2_-VASc score was originally developed for stroke risk prediction in patients with AF (Lip et al., 2010; Camm et al., 2012; January et al., 2014), transportability of the score across different populations has been investigated. One research showed the CHA2DS2-VASc score is useful in predicting poor 12-month outcomes following myocardial infarction in diabetic patients without AF (Hudzik et al., 2016). Moreover, some studies have demonstrated the score achieves high performance for predicting new-onset AF in middle-aged adults and patients with cancer (Saliba et al., 2016; Hu and Lin, 2017; Hu et al., 2019). To our knowledge, the current investigation is the first to assess the predictive role of CHA_2_DS_2_-VASc score for the incidence and HR of AF. Because many of the individual RFs included in the score are also RFs for AF, AF risk increased with increasing CHA_2_DS_2_-VASc score among total patients and GD patients with and without cholecystectomy.

AF is defined as a supraventricular tachyarrhythmia with uncoordinated atrial activation and consequently ineffective atrial contraction (January et al., 2014). Many established and potential RFs for AF induce structural and histopathologic changes in the atrium, which are characterized directly or indirectly by fibrosis, inflammation, and cellular and molecular changes (Staerk et al., 2017). Although the actual pathophysiology through which GD leads to AF remains undetermined, certain observational evidence might offer possible explanations. GD can lead to inflammation that is characterized by bile retention or microbiota in the gallbladder (Lammert et al., 2016). The inflammation can cause oxidative damage in the atrium, which may be followed by AF. Inflammatory biomarkers, such as C-reactive protein and interleukin-6, increase in AF (January et al., 2014; Gutierrez and Van Wagoner, 2015; Harada et al., 2015). Therefore, cholecystectomy is assumed to reduce AF risk through the elimination of the inflammatory mechanism.

**Figure 4:**
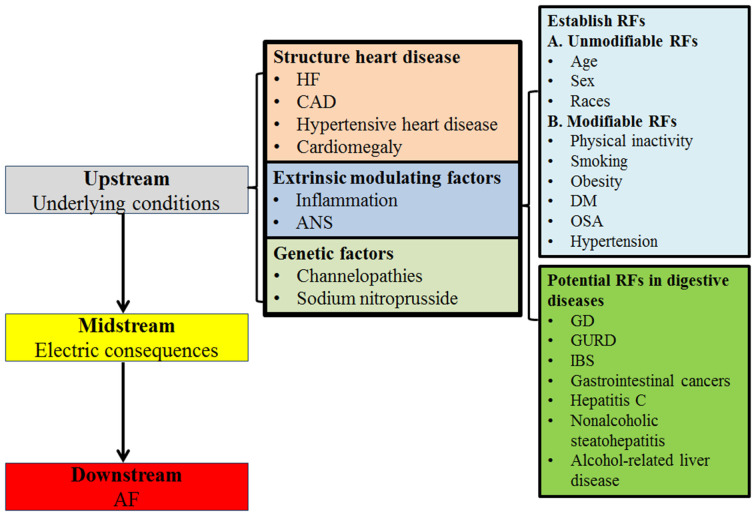
Mechanisms of AF. HF, heart failure; CAD, coronary artery disease; ANS, Autonomic nervous system; RFs, risk factors; DM, diabetes mellitus; OSA, obstructive sleep apnea; GD, gallstone disease; GURD, gastroesophageal reflux disease; IBD, inflammatory bowel disease.

Some of the hepatobiliary and gastrointestinal diseases were independently associated with an increased AF risk. IBD including ulcerative colitis and Crohn’s disease, that are significantly associated with AF incidence (HR = 1.36, 95% CI = 1.20–1.55), could be caused by an inflammatory mechanism (Choi et al., 2019). The incidence and risk of new-onset AF significantly increased, especially in lung and gastrointestinal cancers, possibly due to systemic inflammation (Jakobsen et al., 2019). Liver diseases, including hepatitis C, nonalcoholic steatohepatitis, alcohol-related liver disease, and primary liver cancer, increased the prevalence and incidence of new-onset AF, as they share the underlying link of autonomic dysfunction and inflammation (Huang et al., 2018). Liver cirrhosis increased the risk of AF (HR = 1.46; 95% CI = 1.18–1.80), likely due to cirrhotic cardiomyopathy, vasoactive intestinal peptide, and galectin-3 (Lippi et al., 2015; Xi et al., 2015; Lee et al., 2017; Huang et al., 2018). GERD increased AF risk (HR = 1.31, 95% CI = 1.06–1.61), possibly because of vagal nerve overstimulation, inflammation, autoimmune response, or acid stimulation of the lower esophagus (Huang et al., 2012). Accumulating evidence suggests autonomic dysfunction may contribute to the GD by impairing motility in the gallbladder and sphincter of Oddi (Chawla et al., 2001; Villanacci et al., 2016). Besides inflammatory mechanisms, autonomic dysfunction, which has been observed in patients with GD, may be responsible for the association between GD and an increased risk of AF (Kanoupakis et al., 2000). Figure 4 lists the plausible mechanisms of nonvalvular AF and associating RFs (Iwasaki et al., 2011; Staerk et al., 2017).

Gallstones are classified into cholesterol gallstones (90%) and pigment stones (10%). The mechanism of cholesterol gallstones is caused by disturbance of biliary cholesterol homeostasis. The pathophysiology of pigment gallstones results from hepatic hypersecretion of bilirubin, bile stasis, or bacterial infection (Lammert et al., 2016). The biochemical or inflammatory process caused by abnormal metabolism of cholesterol or bilirubin might induce left atrium remodeling and then induced AF development (Camm et al., 2012). The cholecystectomy might eliminate partially reversible factors of AF progression.

One of the advantages of this study is its use of nationwide population-based data which are highly representative of the general population. However, certain limitations of our study must be considered. First, limited to usage specifications of the NHIRD, the research period of enrollment was from 2001–2009 and the end of follow-up date was up to the end of December 31, 2011. If the research has longer collection and follow-up, the validity should be higher. Second, we did not consider whether patients had undergone open or laparoscopic cholecystectomy. The AF outcome with different procedures warrants further evaluation. Third, the detailed GD subtypes could not be used due to clinical coding habits. Fourth, AF subtypes (paroxysmal, persistent, long-standing persistent, or permanent) could not be classified because of the lack of specific *ICD-9-CM* codes. Fifth, evidence derived from a retrospective cohort study is generally lower in statistical quality than that derived from randomized trials due to potential bias related to adjustments for confounding variables. Sixth, the patient data recorded in the NHIRD are anonymous, and therefore, detailed personal information regarding smoking and sleep habits, body mass index, physical inactivity, lifestyle, or family history could not be obtained, although these are RFs for AF. Changes in lifestyle and body weight may affect the results. Simultaneously, relevant clinical variables, such as symptoms, signs, imaging results, pathology findings, and serum laboratory data, were unavailable in the NHIRD. However, records related to AF, GD, and cholecystectomy diagnoses were highly reliable. Further prospective observational and interventional studies are necessary to support the findings.

This large data-based longitudinal follow-up and retrospective study revealed an association between GD (both asymptomatic and symptomatic) and increased AF risk. Cholecystectomy is related to reduce AF risk. Our findings suggest that gastroenterologists and gastrointestinal surgeons must pay close attention to patients with GD in light of the risk of AF, particularly in patients with conventional stroke RFs.

## Data Availability Statement

The original contributions presented in the study are included in the article, further inquiries can be directed to the corresponding author.

## Ethics Statement

The studies involving human participants were reviewed and approved by Ethics Review Board of China Medical University. The ethics committee waived the requirement of written informed consent for participation.

## Author Contributions

TCH and Y-CC proposed the research idea and wrote the manuscript. C-CL supported the data analysis. H-CT provided the clinical suggestions. C-YW wrote the manuscript and prepared the manuscript for submission. Y-HY and CYH supported the literature review. All authors contributed to the article and approved the submitted version.

## Conflict of Interest

The authors declare that the research was conducted in the absence of any commercial or financial relationships that could be construed as a potential conflict of interest.

## Publisher’s Note

All claims expressed in this article are solely those of the authors and do not necessarily represent those of their affiliated organizations, or those of the publisher, the editors and the reviewers. Any product that may be evaluated in this article, or claim that may be made by its manufacturer, is not guaranteed or endorsed by the publisher.

## References

[B1] Alonso A. Krijthe B. P. Aspelund T. Stepas K. A. Pencina M. J. Moser C. B.. (2013). Simple risk model predicts incidence of atrial fibrillation in a racially and geographically diverse population: the CHARGE-AF consortium. J. Am. Heart Assoc. 2:e000102. 10.1161/JAHA.112.000102 23537808 PMC3647274

[B2] Bortnichak E. A. Freeman D. H. Jr. Ostfeld A. M. Castelli W. P. Kannel W. B. Feinleib M.. (1985). The association between cholesterol cholelithiasis and coronary heart disease in framingham, massachusetts. Am. J. Epidemiol. 121, 19–30. 10.1093/oxfordjournals.aje.a113978 3155483

[B3] Camm A. J. Lip G. Y. De Caterina R. Savelieva I. Atar D. Hohnloser S. H.. (2012). 2012 focused update of the ESC Guidelines for the management of atrial fibrillation: an update of the 2010 ESC Guidelines for the management of atrial fibrillation. developed with the special contribution of the european heart rhythm association. Eur. Heart J. 33, 2719–2747. 10.1093/eurheartj/ehs253 22922413

[B4] Choi Y. J. Choi E. K. Han K. D. Park J. Moon I. Lee E.. (2019). Increased risk of atrial fibrillation in patients with inflammatory bowel disease: a nationwide population-based study. World J. Gastroenterol. 25, 2788–2798. 10.3748/wjg.v25.i22.2788 31236001 PMC6580358

[B5] Chawla A. Puthumana L. Thuluvath P. J. (2001). Autonomic dysfunction and cholelithiasis in patients with cirrhosis. Dig. Dis. Sci. 46, 495–498. 10.1023/a:1005630711669 11318521

[B6] Fairfield C. J. Wigmore S. J. Harrison E. M. (2019). Gallstone disease and the risk of cardiovascular disease. Sci. Rep. 9:5830. 10.1038/s41598-019-42327-2 30967586 PMC6456597

[B7] Fan L. L. Chen B. H. Dai Z. J. (2017). The relation between gallstone disease and cardiovascular disease. Sci. Rep. 7:15104. 10.1038/s41598-017-15430-5 29118437 PMC5678091

[B8] Friedman G. D. (1993). Natural history of asymptomatic and symptomatic gallstones. Am. J. Surg. 165, 399–404. 10.1016/s0002-9610(05)80930-4 8480871

[B9] Friedman G. D. Raviola C. A. Fireman B. (1989). Prognosis of gallstones with mild or no symptoms: 25 years of follow-up in a health maintenance organization. J. Clin. Epidemiol. 42, 127–136. 10.1016/0895-4356(89)90086-3 2918322

[B10] Gutierrez A. Van Wagoner D. R. (2015). Oxidant and inflammatory mechanisms and targeted therapy in atrial fibrillation: an update. J. Cardiovasc. Pharmacol. 66, 523–529. 10.1097/FJC.0000000000000313 26335221 PMC4674316

[B11] Harada M. Van Wagoner D. R. Nattel S. (2015). Role of inflammation in atrial fibrillation pathophysiology and management. Circ. J. 79, 495–502. 10.1253/circj.CJ-15-0138 25746525 PMC4457364

[B12] Hu W. S. Hsieh M. H. Lin C. L. (2019). A novel atrial fibrillation prediction model for Chinese subjects: a nationwide cohort investigation of 682 237 study participants with random forest model. Europace 21, 1307–1312. 10.1093/europace/euz036 31067312

[B13] Hu W. S. Lin C. L. (2017). Comparison of CHA(2)DS(2)-VASc, CHADS(2) and HATCH scores for the prediction of new-onset atrial fibrillation in cancer patients: a nationwide cohort study of 760,339 study participants with competing risk analysis. Atherosclerosis 266, 205–211. 10.1016/j.atherosclerosis.2017.10.007 29049919

[B14] Huang C. C. Chan W. L. Luo J. C. Chen Y. C. Chen T. J. Chung C. M.. (2012). Gastroesophageal reflux disease and atrial fibrillation: a nationwide population-based study. PLoS One 7:e47575. 10.1371/journal.pone.0047575 23077642 PMC3471851

[B15] Huang W. A. Dunipace E. A. Sorg J. M. Vaseghi M. (2018). Liver disease as a predictor of new-onset atrial fibrillation. J. Am. Heart Assoc. 7:e008703. 10.1161/JAHA.118.008703 30371253 PMC6201455

[B16] Hudzik B. Szkodzinski J. Hawranek M. Lekston A. Polonski L. Gąsior M. (2016). CHA2DS2-VASc score is useful in predicting poor 12-month outcomes following myocardial infarction in diabetic patients without atrial fibrillation. Acta Diabetol. 53, 807–815. 10.1007/s00592-016-0877-6 27339195 PMC5014889

[B17] Ibrahim M. Sarvepalli S. Morris-Stiff G. Rizk M. Bhatt A. Walsh R. M.. (2018). Gallstones: Watch and wait, or intervene? Cleve. Clin. J. Med. 85, 323–331. 10.3949/ccjm.85a.17035 29634468

[B18] Iwasaki Y. K. Nishida K. Kato T. Nattel S. (2011). Atrial fibrillation pathophysiology: implications for management. Circulation 124, 2264–2274. 10.1161/CIRCULATIONAHA.111.019893 22083148

[B19] Jakobsen C. B. Lamberts M. Carlson N. Lock-Hansen M. Torp-Pedersen C. Gislason G. H.. (2019). Incidence of atrial fibrillation in different major cancer subtypes: a Nationwide population-based 12 year follow up study. BMC Cancer 19:1105. 10.1186/s12885-019-6314-9 31726997 PMC6854796

[B20] January C. T. Wann L. S. Alpert J. S. Calkins H. Cigarroa J. E. Cleveland J. C.. (2014). 2014 AHA/ACC/HRS guideline for the management of patients with atrial fibrillation: a report of the american college of cardiology/american heart association task force on practice guidelines and the heart rhythm society. Circulation 130, e199–e267. 10.1161/CIR.0000000000000041 24682347 PMC4676081

[B21] Jiang Z. Y. Sheng X. Xu C. Y. Li W. W. Chang X. X. Sun L. Y.. (2013). Gallbladder gallstone disease is associated with newly diagnosed coronary artery atherosclerotic disease: a cross-sectional study. PLoS One 8:e75400. 10.1371/journal.pone.0075400 24058685 PMC3776774

[B22] Kannel W. B. Benjamin E. J. (2009). Current perceptions of the epidemiology of atrial fibrillation. Cardiol. Clin. 27, vii13–vii24. 10.1016/j.ccl.2008.09.015 19111760 PMC2917063

[B23] Kanoupakis E. M. Manios E. G. Mavrakis H. E. Kaleboubas M. D. Parthenakis F. I. Vardas P. E. (2000). Relation of autonomic modulation to recurrence of atrial fibrillation following cardioversion. Am. J. Cardiol. 86, 954–958. 10.1016/s0002-9149(00)01129-2 11053706

[B24] Lammert F. Gurusamy K. Ko C. W. Miquel J. F. Méndez-Sánchez N. Portincasa P.. (2016). Gallstones. Nat. Rev. Dis. Primers 2:16024. 10.1038/nrdp.2016.24 27121416

[B25] Lee H. Choi E. K. Rhee T. M. Lee S. R. Lim W. H. Kang S. H.. (2017). Cirrhosis is a risk factor for atrial fibrillation: a nationwide, population-based study. Liver Int. 37, 1660–1667. 10.1111/liv.13459 28432810

[B26] Lip G. Y. H. Nieuwlaat R. Pisters R. Lane D. A. Crijns H. J. G. M. (2010). Refining clinical risk stratification for predicting stroke and thromboembolism in atrial fibrillation using a novel risk factor-based approach: the euro heart survey on atrial fibrillation. Chest 137, 263–272. 10.1378/chest.09-1584 19762550

[B27] Lippi G. Cervellin G. Sanchis-Gomar F. (2015). Galectin-3 in atrial fibrillation: Simple bystander, player or both? Clin. Biochem. 48, 818–822. 10.1016/j.clinbiochem.2015.04.021 25952321

[B28] Lv J. Qi L. Yu C. Guo Y. Bian Z. Chen Y.. (2015). Gallstone disease and the risk of ischemic heart disease. Arterioscler. Thromb. Vasc. Biol. 35, 2232–2237. 10.1161/ATVBAHA.115.306043 26272939 PMC4587542

[B29] Marcus G. M. Alonso A. Peralta C. A. Lettre G. Vittinghoff E. Lubitz S. A.. (2010). European ancestry as a risk factor for atrial fibrillation in African Americans. Circulation 122, 2009–2015. 10.1161/CIRCULATIONAHA.110.958306 21098467 PMC3058884

[B30] Olaiya M. T. Chiou H. Y. Jeng J. S. Lien L. M. Hsieh F. I. (2013). Significantly increased risk of cardiovascular disease among patients with gallstone disease: a population-based cohort study. PLoS One 8:e76448. 10.1371/journal.pone.0076448 24098504 PMC3789705

[B31] Page R. L. Wilkinson W. E. Clair W. K. McCarthy E. A. Pritchett E. L. (1994). Asymptomatic arrhythmias in patients with symptomatic paroxysmal atrial fibrillation and paroxysmal supraventricular tachycardia. Circulation 89, 224–227. 10.1161/01.cir.89.1.224 8281651

[B32] Reynolds M. R. Lavelle T. Essebag V. Cohen D. J. Zimetbaum P. (2006). Influence of age, sex and atrial fibrillation recurrence on quality of life outcomes in a population of patients with new-onset atrial fibrillation: the fibrillation registry assessing costs, therapies, adverse events and lifestyle (FRACTAL) study. Am. Heart J. 152, 1097–1103. 10.1016/j.ahj.2006.08.011 17161061 PMC1820843

[B33] Rodriguez C. J. Soliman E. Z. Alonso A. Swett K. Okin P. M. Goff D. C.. (2015). Atrial fibrillation incidence and risk factors in relation to race-ethnicity and the population attributable fraction of atrial fibrillation risk factors: the multi-ethnic study of atherosclerosis. Ann. Epidemiol. 25, 71–76. 10.1016/j.annepidem.2014.11.024 25523897 PMC4559265

[B34] Saliba W. Gronich N. Barnett-Griness O. Rennert G. (2016). Usefulness of CHADS2 and CHA2DS2-VASc scores in the prediction of new-onset atrial fibrillation: a population-based study. Am. J. Med. 129, 843–849. 10.1016/j.amjmed.2016.02.029 27012854

[B35] Schnabel R. B. Yin X. Gona P. Larson M. G. Beiser A. S. McManus D. D.. (2015). 50 year trends in atrial fibrillation prevalence, incidence, risk factors and mortality in the Framingham Heart Study: a cohort study. Lancet 386, 154–162. 10.1016/S0140-6736(14)61774-8 25960110 PMC4553037

[B36] Staerk L. Sherer J. A. Ko D. Benjamin E. J. Helm R. H. (2017). Atrial fibrillation: epidemiology, pathophysiology and clinical outcomes. Circ. Res. 120, 1501–1517. 10.1161/CIRCRESAHA.117.309732 28450367 PMC5500874

[B37] Upala S. Sanguankeo A. Jaruvongvanich V. (2017). Gallstone disease and the risk of cardiovascular disease: a systematic review and meta-analysis of observational studies. Scand. J. Surg. 106, 21–27. 10.1177/1457496916650998 27255283

[B38] Villanacci V. Del Sordo R. Salemme M. Cadei M. Sidoni A. Bassotti G. (2016). The enteric nervous system in patients with calculous and acalculous gallbladder. Dig. Liver Dis. 48, 792–795. 10.1016/j.dld.2016.03.014 27068404

[B39] Wei C. Y. Chuang S. H. Lin C. L. Kung W. M. Tai H. C. Tsai K. W.. (2019). Reduced risk of stroke following cholecystectomy: a nationwide population-based study. J. Gastroenterol. Hepatol. 34, 1992–1998. 10.1111/jgh.14678 31165511

[B40] Wei C. Y. Chung T. C. Chen C. H. Lin C. C. Sung F. C. Chung W. T.. (2014). Gallstone disease and the risk of stroke: a nationwide population-based study. J. Stroke Cerebrovasc. Dis. 23, 1813–1820. 10.1016/j.jstrokecerebrovasdis.2014.04.024 24957305

[B41] Wirth J. di Giuseppe R. Wientzek A. Katzke V. A. Kloss M. Kaaks R.. (2015). Presence of gallstones and the risk of cardiovascular diseases: the EPIC-Germany cohort study. Eur. J. Prev. Cardiol. 22, 326–334. 10.1177/2047487313512218 24177267

[B42] Xi Y. James Chao Z. Y. Yan W. Abbasi S. Yin X. Mathuria N.. (2015). Neuronally released vasoactive intestinal polypeptide alters atrial electrophysiological properties and may promote atrial fibrillation. Heart Rhythm 12, 1352–1361. 10.1016/j.hrthm.2015.03.003 25748673 PMC4684179

[B43] Zheng Y. Xu M. Heianza Y. Ma W. Wang T. Sun D.. (2018). Gallstone disease and increased risk of mortality: Two large prospective studies in US men and women. J. Gastroenterol. Hepatol. 33, 1925–1931. 10.1111/jgh.14264 29671893 PMC9015210

[B44] Zheng Y. Xu M. Li Y. Hruby A. Rimm E. B. Hu F. B.. (2016). Gallstones and risk of coronary heart disease: prospective analysis of 270 000 men and women from 3 US cohorts and meta-analysis. Arterioscler. Thromb. Vasc. Biol. 36, 1997–2003. 10.1161/ATVBAHA.116.307507 27540264 PMC5001914

[B45] Zimetbaum P. (2017). Atrial fibrillation. Ann. Intern. Med. 166, ITC33–ITC48. 10.7326/AITC201703070 28265666

